# Supplementary material for: High-throughput 96-well plate-based porcine antibody isolation protocol
Source: PLoS One. 2025 Mar 27;20(3):e0320501. doi: 10.1371/journal.pone.0320501 (PMC11949346; doi:10.1371/journal.pone.0320501)
Supplement: S1 File — (PDF) [file pone.0320501.s001.pdf]

High-throughput 96-well plate-based porcine antibody isolation protocol

RESERVED DOI:
10.17504/protocols.io.yxmvmee6ng3p/v1

John Byrne[1], Christina Bourne[1], Sitka Eguiluz[1], Stephanie Langel[2], Elisa Crisci[1]

[1]Department of Population Health and Pathobiology, North Carolina College of Veterinary Medicine, Raleigh, NC;
[2]Center for Global Health and Diseases, Department of Pathology, Case Western Reserve University School of Medicine, Cleveland, OH

J John Byrne
Department of Population Health and Pathobiology, North Caro...

Protocol Info: John Byrne, Christina Bourne, Sitka Eguiluz, Stephanie Langel, Elisa Crisci . High-throughput 96-well plate-based porcine antibody isolation protocol. **protocols.io** **https://protocols.io/view/high-throughput-96-well-plate-based-porcine-antibo-de6c3haw**

Created: June 05, 2024

Last Modified: February 21, 2025

Protocol Integer ID: 101284

Keywords: Antibody, Pig, Swine, Porcine, Resin, Chromatography, IgA, IgG, Isolation, High Throughput

Funders Acknowledgements:
Bill and Melinda Gates Foundation
Grant ID: INV-22595D

Disclaimer

DISCLAIMER – FOR INFORMATIONAL PURPOSES ONLY; USE AT YOUR OWN RISK

The protocol content here is for informational purposes only and does not constitute legal, medical, clinical, or safety advice, or otherwise; content added to **protocols.io** is not peer reviewed and may not have undergone a formal approval of any kind. Information presented in this protocol should not substitute for independent professional judgment, advice, diagnosis, or treatment. Any action you take or refrain from taking using or relying upon the information presented here is strictly at your own risk. You agree that neither the Company nor any of the authors, contributors, administrators, or anyone else associated with **protocols.io**, can be held responsible for your use of the information contained in or linked to this protocol or any of our Sites/Apps and Services.

Abstract

This protocol describes a modified column chromatography technique to isolate both IgG and IgA from pregnant/lactating swine to use in a variety of immunoassays. This high throughput method allows for both a rapid and consistent isotype yield from individual animals and different tissue samples.

Materials

Glycine, 0.2M buffer soln., pH 2.5 Cat# J61855.AP (Thermo Scientific)
Tris Hydrochloride, 1M Solution (pH 8.0/Mol. Biol.) Cat# BP1758-500 (Fisher Scientific)
Ethanol, Absolute (200 Proof), Molecular Biology Grade Cat# BP2818500 (Fisher Scientific)
Tris-EDTA, 1x Solution, pH 8.0 ± 0.1 Cat# 77-86-1 (Fisher Scientific)
Sodium chloride, ACS, 99.0% min Cat# 7647-14-5 (Fisher Scientific)
Pierce Protein G Agarose Cat# 20399 (Thermo Scientific)
Peptide M / Agarose Cat# gel-pdm-5 (InvivoGen)
CaptureSelect IgA-XL Affinity Matrix Cat# 2943972010 (Thermo Scientific)
Ultra-pure water - In House
Corning Mediatech Cell Culture Phosphate Buffered Saline (10X) Cat# MT-46013CM (Fisher Scientific)
Universal 200ul Pipette tips Cat# 76322-144 (VWR)
BrandTech Scientific 781722, 96-Well Plates, immunoGrade, Clear Transparent, Flat Bottom Cat# 91-415F (Genesee Scientific)
Multiscreen 96 well Plate, hydrophobic PVDF membrane Cat# MSIPS4W10 (Millipore Sigma)
Thermo Scientific NanoDrop 2000c Spectrophotometer
Sorvall Legend XTR (Refrigerated), 120V (Thermo Scientific)
Sorvall Legend Micro 21R Microcentrifuge (Thermo Scientific)

Before start

Prepare 1x PBS

Prepare 0.1M Glycine

Prepare 70% ETOH

Prepare Ab Binding Buffer (10mM TRIS, 1mM EDTA, 300mM NaCl)

Sample Preparation

1m

1 Quickly vortex all samples to ensure no clotting from serum and no fat blockages from milk

2 Centrifuge all samples at Room temperature for 2000 x g, Room temperature, 00:01:00

1m

3 Add 100 µL sample (serum/milk/colostrum) to 100 µL Ab Binding Buffer
- DO NOT ADD ANY PRECIPITATE TO THE MIX

Resin Plate Preparation

3m

4 Remove the Multiscreen 96 well ELISpot Plate from packaging

5 Place the Multiscreen 96 well ELISpot Plate on top of a 96-Well immunoGrade ELISA Plate ensuring the ejection ports of the ELISpot plate are in line with the ELISA plate wells

6 Add 50 µL Protein G, CaptureSelect IgA, or Peptide M to each well
- ONLY USE ONE RESIN PER WELL

7 Wash all wells by adding 50 µL 70% ETOH to each well

8 Centrifuge the plate stack at 4000 x g, Room temperature, 00:03:00

3m

9 Discard flow through from the bottom plate

10 Wash all wells by adding 200 µL 1x 7.4pH PBS to each well

11 Centrifuge the plate stack at 4000 x g, Room temperature, 00:03:00

3m

12 Discard flow through from the bottom plate

13 Restack the plates

Antibody Isolation

41m

14 Add 100 µL Sample (serum/milk/colostrum) / Ab Binding Buffer mix per well to each plate

15 Place the plate on a plate shaker and shake 600 rpm, Room temperature , 00:30:00

30m

16 Remove the plate stack from the shaker and centrifuge the plate at 4000 x g, Room temperature, 00:03:00

3m

17 Discard the flow through from the bottom plate

18 Restack the plates

19 Wash all wells by adding 200 µL Ab Binding Buffer to each well

20 Centrifuge the plate stack at 4000 x g, Room temperature, 00:03:00

3m

21 Discard the flow through from the bottom plate

22 Repeat Steps 19-21 3 times

23 Place a new 96-Well immunoGrade ELISA Plate under the Multiscreen 96 well ELISpot Plate to ensure that collected antibody are pure

24 Add 100 µL 0.1M Glycine and incubate 00:02:00 , Room temperature

2m

25 Elute the antibodies by centrifuging the plate stack at 4000 x g, Room temperature, 00:03:00 — 3m

26 Repeat step 24 and 25

27 Add 20 µL 1M TRIS to each well to neutralize the solution

Antibody Concentration and Storage — 11m

28 Label one Amicon ultra 0.5 50k Centrifuge filter for each sample

29 Remove the 220 µL glycine-TRIS solution from the well and place into the corresponding Amicon ultra 0.5 50k Centrifuge filter

30 Centrifuge the filter tubes at 12000 x g, Room temperature, 00:05:00 — 5m

31 Wash each sample with 400 µL 1x 7.4pH PBS

32 Centrifuge the filter tubes at 12000 x g, Room temperature, 00:05:00 — 5m

33 Label one Amicon ultra 0.5 50k Centrifuge collection tube to store each sample

34 Invert the Amicon ultra 0.5 50k Centrifuge filter into the new labeled storage tube

35 Centrifuge the filter tubes at 1000 x g, Room temperature, 00:01:00 — 1m
- this should yield ~ 100ul of isolated antibody

36 Remove the filter from each tube and store isolated antibodies at 4 °C until later use

Quantify Antibody concentration

37 Use NanoDrop, select the "Protein A280" program

38 Pipette 1 µL isolated antibody on the NanoDrop pedestal

39 Analyze the sample

40 Record published mg/mL
